# EPIGENETIC AGING OF THE DEMOGRAPHICALLY NONAGING NAKED MOLE RAT

**DOI:** 10.1093/geroni/igac059.1731

**Published:** 2022-12-20

**Authors:** Csaba Kerepesi

**Affiliations:** Institute for Computer Science and Control (SZTAKI), Budapest, Budapest, Hungary

## Abstract

The naked mole-rat (NMR) is an exceptionally long-lived rodent that shows no increase of mortality with age, defining it as a demographically non-aging mammal. Here, we perform bisulfite sequencing of the blood of>100 NMRs, assessing>3 million common CpG sites. Unsupervised clustering based on sites whose methylation correlates with age reveals an age-related methylome remodeling, and we also observe a methylome information loss, suggesting that NMRs age. We develop an epigenetic aging clock that accurately predicts the NMR age. We show that these animals age much slower than mice and much faster than humans, consistent with their known maximum lifespans. Interestingly, patterns of age-related changes of clock sites in Tert and Prpf19 differ between NMRs and mice, but there are also sites conserved between the two species. Together, the data indicate that NMRs, like other mammals, epigenetically age even in the absence of demographic aging of this species.

